# Enzyme immobilization in hydrogels: A perfect liaison for efficient and sustainable biocatalysis

**DOI:** 10.1002/elsc.202100087

**Published:** 2021-12-21

**Authors:** Johanna Meyer, Lars‐Erik Meyer, Selin Kara

**Affiliations:** ^1^ Institute of Technical Chemistry Leibniz University Hannover Hannover Germany; ^2^ Biocatalysis and Bioprocessing Group Department of Biological and Chemical Engineering Aarhus University Aarhus Denmark

**Keywords:** biocatalysis, enzyme immobilization, hydrogels, process intensification, sustainable synthesis

## Abstract

Biocatalysis is an established chemical synthesis technology that has by no means been restricted to research laboratories. The use of enzymes for organic synthesis has evolved greatly from early development to proof‐of‐concept – from small batch production to industrial scale. Different enzyme immobilization strategies contributed to this success story. Recently, the use of hydrogel materials for the immobilization of enzymes has been attracting great interest. Within this review, we pay special attention to recent developments in this key emerging field of research. Firstly, we will briefly introduce the concepts of both biocatalysis and hydrogel worlds. Then, we list recent interesting publications that link both concepts. Finally, we provide an outlook and comment on future perspectives of further exploration of enzyme immobilization strategies in hydrogels.

**Abbreviations**
HEMA: 2‐hydroxyethyl methacrylate
HRP: horseradish peroxidase
NAD[P]H: nicotinamide adenine dinucleotide [phosphate]
PEG: polyethylene glycol
PEGDA: polyethylene glycol diacrylate
pILs: polymerized ionic liquids
STY: space time yield.

## 1 INTRODUCTION

For more than 100 years, enzymatic catalysis has made its way into modern organic chemistry. Nowadays, biocatalysis is a powerful tool for selective organic synthesis [1, 2, 3, 4, 5], and publications from the last three years cover the whole area of enzymatic synthesis and provide a comprehensive overview [6, 7, 8, 9, 10, 11, 12, 13, 14, 15, 16, 17, 18, 19]. Applied either as whole (resting) cells or as purified enzymes, a biocatalyzed reaction generally benefits from (i) exclusive chemo‐, stereo‐, and/or enantioselectivities, (ii) mild reaction conditions, (iii) a broad substrate spectrum, and (iv) its low environmental impact since enzymes are biodegradable. On the other hand, certain critical aspects must be considered when enzyme‐catalyzed reactions are used for organic synthesis: (i) A narrow window of operation parameters (pH, temperature or inhibitory effects), (ii) enzyme's low tolerance to organic solvents, and/or (iii) the use of expensive cofactors like nicotinamide adenine dinucleotide [phosphate] (NAD[P]H) that are needed at stoichiometric amounts [20].

To overcome these challenges, modern molecular biological tools have been effectively used to optimize enzymes’ operational window for broader pH and temperature ranges as well as to increase their stability in organic media [21]. For the use of NAD[P]H, new cofactor regeneration methods have been continuously developed [22, 23] or their mimics can also be applied [24, 25]. Although these approaches have been beneficial to deal with the aforementioned limitations, further optimizations to reach high volumetric productivities (mass_product_⋅volume_reaction_^–1^⋅time_reaction_^–1^) at technical scales can be possible with process engineering. Hence, researchers began to include and integrate medium engineering‐ [26, 27, 28, 29] and reaction engineering [30, 31] into enzymatic synthesis targeting these drawbacks from all possible directions. Examples of medium engineering include (but not limited to) the use of non‐conventional media like solvent‐free systems (so‐called ‘neat substrates’) [32, 33], organic media [34, 35, 36], ionic liquids [37, 38, 39], or deep eutectic solvents [40, 41, 42, 43, 44, 45]. Whereas, selected examples of reaction engineering include flow biocatalysis [46], improving the downstream processing [47, 48], or in situ product removal techniques [49]. Researchers have also been focusing on enzyme immobilization in regards to bioprocess development [50, 51]. Here, the biocatalyst benefits not only from higher stability, but also from broader applicability with respect to a simpler recycling of the catalyst, from enzyme‐free product streams, and by enabling the use of enzymes in continuous operations [52].

Different forms of immobilization were described in the literature, and overall, three types of enzyme immobilization are generally known: (i) Binding the enzyme to a (porous) support, (ii) crosslinking of the enzyme, and (iii) encapsulation/entrapment of the biocatalyst into a matrix [53, 54]. Diverse immobilization methods as well as different carrier materials have already been described to enable high immobilization and activity yields combined with mechanical stability of the materials.

Besides the aforementioned methods for enzyme immobilization, the use of hydrogels for heterogenization of enzymes has attracted great attention in the last few years. An unbeatable argument compared to other immobilization materials is the fact that hydrogels, per se, contain water: Hydrogels are referred to as polymeric materials that are capable of absorbing a large amount of water. For most of the enzymes in non‐aqueous media, hydrogels a prerequisite that can provide the desired microenvironment for enzymes which is highly advantageous. Here, it is worth mentioning that enzyme immobilization in gels has been pioneered by Manfred Reetz with his great contribution since end of 90s [55, 56, 57, 58].

**FIGURE 1:**
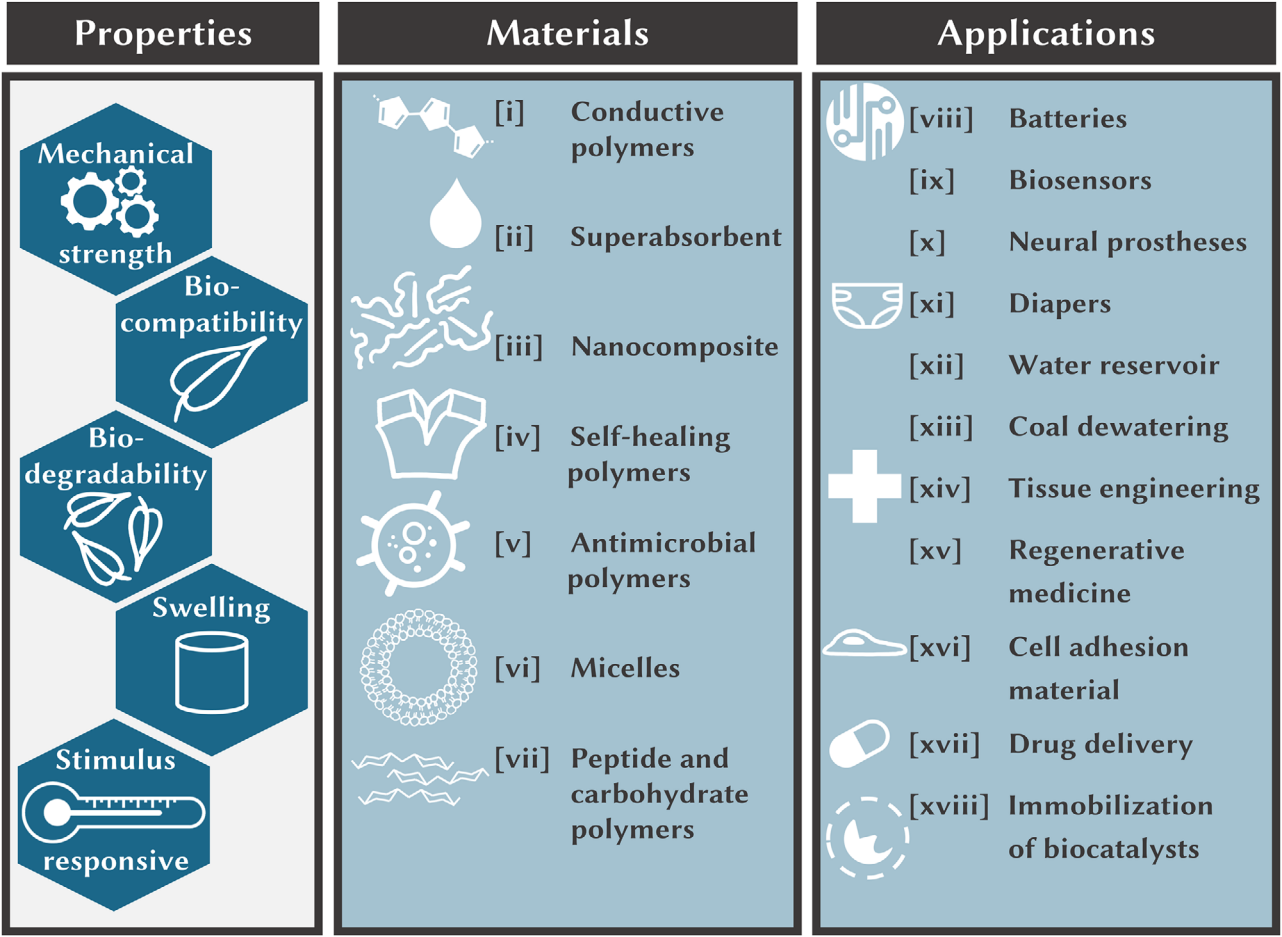
Overview of properties, materials, and applications of hydrogels. References: [i] = [123, 124, 125]; [ii] = [126, 127, 128, 129]; [iii] = [130]; [iv] = [131, 132]; [v] = [133, 134]; [vi] = [135, 136]; [vii] = [137, 138, 139]; [viii] = [140, 141]; [ix] = [142, 143]; [x] = [144]; [xi] = [145]; [xii] = [70, 146, 147]; [xiii] = [148]; [xiv] = [149, 150, 151]; [xv] = [152, 153]; [xvi] = [154, 155]; [xvii] = [156, 157, 158, 159]; [xviii] = covered within this review

Hydrogels, as we understand the term within this publication, consist of a crosslinked polymer network. Generally, these polymers can absorb immense amounts of water, enlarging their volume and their whole mass while retaining their three‐dimensional shape. The mass fraction of the hydrogels does not change. Often, upon swelling, hydrogels retain their stiffness and therefore, their durability [39]. The synthesis and characterization of hydrogels is a broad research field. Since this review article covers the immobilization of enzymes within hydrogel materials, we would like to refer readers interested in materials preparation to recent review articles [59, 60, 61]. However, we also would like to give a general overview of hydrogel materials and applications to emphasize their broad application fields. A wide range of applications can be found for these polymers, since many hydrogels are described as (i) biodegradable, (ii) biocompatible, (iii) stimuli responsive [62], (iv) antibacterial [63], as well as (v) mechanically strong or even (vi) flexible [64, 65].The application include (i) various facets of the medical field (e.g., implants for knee surgeries [66] or drug delivery systems [67]), (ii) areas in biotechnology, (iii) as superconductors [68], (iv) as immobilization matrix for biocatalysts [69], and (v) on agricultural farms as water storage [70] (see Figure 1 for further details and insights).

Although the technique of enzyme immobilization has long been known, as well as the knowledge of natural and synthetic hydrogels, we are not aware of any review article that explicitly reports on the immobilization of enzymes in hydrogels. It should be noted, however, that often a different terminology is used. For example, we would like to mention the recently published review article by Nöth et al. dealing with the synthesis, concepts, and emerging applications of biocatalytic microgels (μ‐Gel*zymes*) [71]. Additionally, we would like to mention the publication by Zhu et al. reviewing microfluidic immobilized enzyme reactors for continuous biocatalysis providing a broader overview of the application of enzyme immobilization in hydrogel materials focusing on materials for microfluidic reactors [72].

In this review, we aim to present developments in the field of enzyme immobilization in hydrogels published in the last twenty years. In Section 2.1, we focus on the nature of hydrogel materials used for enzyme immobilization in general. In Section 2.2, we address the literature from the last five years with respect to the applications of hydrogel‐based immobilized enzymes.

## 2 ENZYME IMMOBILIZATION IN HYDROGEL MATERIALS

At the beginning of every immobilization procedure there is one big question: Which immobilization carrier and which technique is the most suitable for the biocatalyst and the desired reaction? To answer this question, there are further criteria to be considered; like biocompatibility, product separation, high enzyme loading, biodegradability as well as mechanical and chemical stability [73]. For industrial applications, the cost‐benefit factor plays an important role, and consequently the carrier material should be cheap and easy to produce [74, 75, 76].

### 2.1 Hydrogel materials as enzyme immobilization carriers: Overview, advantages, and challenges

Natural polymers are de quo composed of mostly polysaccharides (e.g., agarose, alginate, carrageenan, cellulose, chitosan, hyaluronic acid, and starch) or proteins (e.g., collagen, fibrin, and gelatin) [77, 78, 79]. The advantage of these materials is that they are inherently biocompatible, are non‐toxic, biodegradable, mechanically flexible, and renewable. For example, alginate, a water‐soluble, naturally occurring anionic polysaccharide, has low production costs and gelation occurs under very mild conditions by adding divalent cations like Ca^2+^, Mn^2+^, or Ba^2+^. Although sodium alginate is commercially available in a wide range of molecular weights (from 32,000 to 400,000 g·mol^−1^) at low prices, an alginate solution with higher molecular weight suffers from extremely high viscosity (∼700 mPa⋅s at an alginate concentration of 35 g⋅L^–1^ [80], compared to 1.0 mPa⋅s of water, each at 20°C). For example, Kucharzyk et al. reported a calcium‐alginate encapsulated laccase from *Cerrena unicolor* 303 for the biodegradation of crude and weathered oil. Additional to the higher activity, the immobilized enzyme showed an increased tolerance to temperature and pH variation if compared to the free enzyme [81, 82]. This enhanced process stability, storability, pH and temperature resistance is reported in many studies, suggesting that the alginate beads or capsules function as a physical barrier protecting the encapsulated enzyme [83, 84, 85, 86]. Although we are aware researchers’ interest in the field of the mechanisms of enzyme immobilization in hydrogels, to the best of our knowledge, there are no specific reports that have been published so far. Enzyme immobilization in hydrogels may be based on encapsulation alone without covalent interactions, but this is associated with the risk of enzyme leaching. To reduce this risk, covalent interactions can be established between the enzyme surface and the hydrogel matrix. This covers the functional groups such as amino, aldehyde, carboxyl and epoxy groups in cellulose, agarose and dextran [87, 88]. Other challenges like a structural inhomogeneity of natural polysaccharides, a lack of gelation control as well as pre‐gelling and post‐gelling limitations must be mentioned here as well [89]. To overcome these challenges, semi‐synthetic polymers were developed. These matrices were formed by a combination of natural building blocks and another synthetic polymer. Additionally, fully synthetic polymers are promising candidates. These materials could provide better material stiffness and enzyme‐ or cell‐matrix interactions compared to natural polymers only. Polyvinyl alcohol (polyPVA)‐based carriers are known as particles with considerable chemical and mechanical stability providing an alternative to overcome the drawbacks of natural polymers [90]. Hydrophilic polymers like polyHEMA which have a poor mechanical stability, possess risks for enzyme leaching and can swell in aqueous media. Therefore, these hydrophilic monomers are usually co‐polymerized with hydrophobic monomers such as methyl methacrylate (MAA) for controlling their degree of swelling and mechanical strength [91]. Another method to reduce the enzyme leaching is the utilization of crosslinkers like poly(ethylene glycol) diacrylate (PEGDA). PEG is a non‐degradable, hydrophilic and biocompatible polymer, while the latter is caused by its low adhesiveness to proteins and cells. It can be acrylated to PEGDA and applied to polymerize highly crosslinked hydrogel networks. PEG and PEGDA are highly water soluble and photopolymerizable at mild conditions avoiding any treatment with heat or organic solvents during the immobilization process. PEGDA polymers are relatively easy to control during polymerization, and adjustable in their microporosity due to a wide range of molecular weights with increasing chain length. These allow for improved mass transport. Choi et al. entrapped a glucose oxidase in hydrogels with a higher molecular weight of PEGDA, leading to less crosslinked hydrogels, a higher water content, a larger mesh size and therefore a better mass transfer of the substrate. Although the glucose oxidase maintained the activity without leakage over a week, however, the hydrogels showed weak mechanical stability [81].

To improve further characteristics of the immobilization matrix, additional monomers and crosslinkers can be introduced. A frequently applied method circumventing mass transport limitations is the covalent surface immobilization of enzymes on polymers by using spacers [92, 93]. Ayhan et al. reported about non‐porous polyPEGDA‐HEMA beads modified with the spacer hexamethylene diamine. The immobilized urea aminohydrolase maintained 73% of their activity for 75 days of repeated use. Besides this major advantage, the rate‐controlling step of the observed process was found to be the enzymatic reaction, because of the low observable Thiele modulus (*ø *= observed reaction rate/mass transfer rate) [94]. In general, significantly low observable Thiele modulus values (*ø ≤* 0.3) [95] mean sufficiently high mass transfer rates having a better chance of supplying substrate to the surface where catalysis takes place compared to reaction rate.

Interesting new features in the field of enzyme immobilization are polymerized ionic liquids (pILs) and polyelectrolytes and some of these can be referred to as hydrogels as well. Nakashima et al. reported a horseradish peroxidase (HRP) encapsulated in pILs‐microparticles being easily recovered by centrifugation of the reaction mixture. The pILs‐microparticles were obtained by emulsion polymerization of 1‐vinyl‐3‐ethylimidazolium bromide (VEtImBr) with the crosslinker N,N’‐methylene bisacrylamide (MBAA). The HRP was modified with a comb‐shaped PEG, allowing multivalent carboxylic anhydride groups to react with the amino groups of the enzyme. By combining the pILs‐microparticles and HRP‐PEG via free‐radical polymerization, the enzyme could be immobilized and possess higher activity compared to conventional polyacrylamide microparticles. The authors state, that this effect was generated by the flexible spacer arm groups. This allows the polymer surface to conform to the structure of the enzyme, thereby preserving and stabilizing the native catalytic activity to a greater extent [96].

To summarize this section, enzymes can be immobilized in/on a large variety of polymeric carriers/matrices. The degree of swelling in aqueous media has an enormous impact on the effectiveness of the immobilization. For most of the enzymes, a high water content is associated with a desired high enzyme activity. However, it also often leads to increased leaching of the enzyme from the polymer matrix. To find the optimum of immobilization yield and activity, co‐polymerizations with other hydrophobic monomers or other immobilization methods such as covalent bonding can be used.

### 2.2 Applications of hydrogel‐based immobilized enzymes

In this section, we will describe recent developments in the field of biocatalyst immobilization in hydrogels for enzymatic synthesis and devote detailed focus on their implementation in special applications.

**FIGURE 2:**
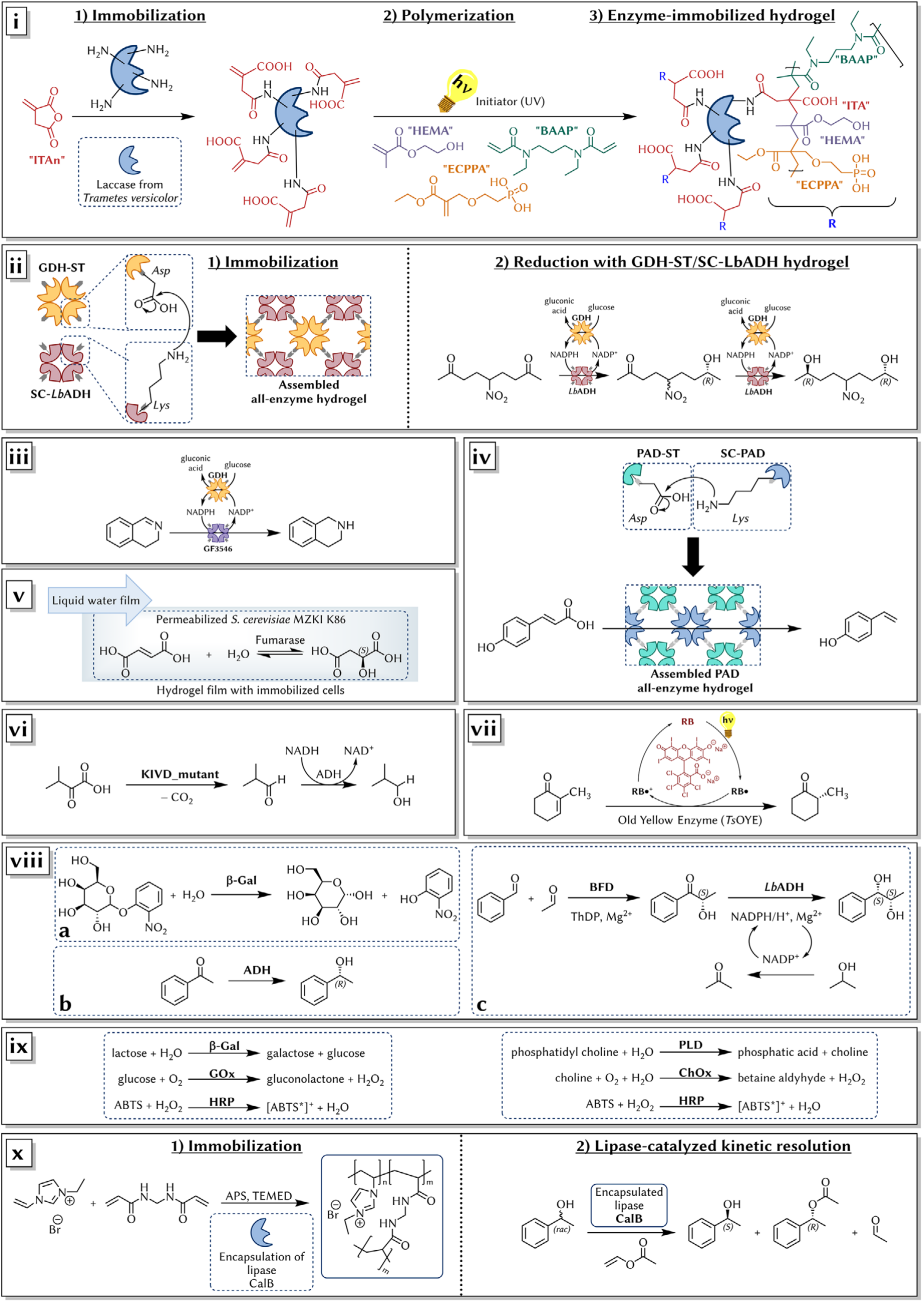
Various applications of hydrogel‐based immobilized enzymes. (i) Synthetic polymer hydrogels for the immobilization of laccase from *Trametes versicolor*. (ii) Protein hydrogel as self‐assembled all‐enzyme hydrogel (left) and its application for reduction (right). (iii) All‐enzyme hydrogel for the reduction of an imine to the corresponding amine coupled with cofactor regeneration. (iv) Biocatalytic synthesis of *para*‐hydroxystyrene from *para*‐coumaric acid via decarboxylation with phenolic acid decarboxylases (PAD). (v) Fumarase‐catalyzed conversion of fumaric acid into l‐malic acid within a two‐plate microreactor equipped with hydrogel‐immobilized yeast cells. (vi) Enzymatic cascade with 3D‐printed enzymes in agarose producing isobutanol in continuous operation. (vii) Reduction of 2‐methylcyclohexenone into (*R*)‐2‐methylcyclohexanone through photosensitization of RB in an alginate hydrogel. (viii) (a) Hydrolysis of *ortho*‐nitrophenyl‐β‐d‐galactopyranoside by β‐Gal, (b) enantioselective reduction of acetophenone by ADH and a two‐step cascade of BFD, and (c) ADH forming (1*S*, 2*S*)‐1‐phenylpropane‐1,2‐diol from benzaldehyde and acetaldehyde. (ix) Two separate trienzymatic cascade reactions in continuous flow producing ABTS. (x) Kinetic resolution of *rac*‐1‐phenylethanol with vinyl acetate and hydrogel‐entrapped CalB as catalyst

Horn et al. used synthetic polymer hydrogels composed out of HEMA, itaconic acid (ITA) and 2‐((2‐(ethoxycarbonyl)prop‐2‐en‐1‐yl)oxy)‐ethyl phosphonic acid (ECPPA), to immobilize a laccase for wastewater treatment [97]. N,N’‐diethyl‐1,3‐bis‐(acrylamido)propane (BAAP) was used as the crosslinker for UV‐initiated radical polymerization and pre modified laccase from *Trametes versicolor* served as biocatalyst. The enzyme‐immobilized hydrogels were synthesized in different (co‐)polymer/crosslinker compositions and used as granules as well as coatings on porous aluminum (Figure 2(i)). Different organic molecules being model components for analgesics, biocides and base materials and softeners of polymer, were degraded with the immobilized laccase with high degradation efficiencies.

Peschke et al. reported on self‐assembling all‐enzyme hydrogels [98]. The authors fused a (*R*)‐selective alcohol dehydrogenase from *Lactobacillus brevis* (*Lb*ADH) and a NADPH‐regenerating glucose 1‐dehydrogenase GDH from *Bacillus subtilis* yielding a hydrogel whose mass consists of 77% enzymes. The SpyTag peptide/SpyCatcher protein (ST/SC) system together with a hexahistidin (His) tag were used to promote the enzyme gelation via crosslinking of the two enzyme building blocks (Figure 2(ii), left). This highly efficient autocatalytic bioconjugation system which forms under physiological conditions is based on the rapid formation of a covalent isopeptide bond through the SpyTag‐SpyCatcher complex [99, 100, 101, 102]. A hydrogel‐containing microfluidic polydimethylsiloxane (PDMS) chip reactor with a 150 μL inner volume was constructed and four different substrates (5‐nitrononane‐2,8‐dione (see also Figure 2(ii), right), acetophenone, 4′‐chloroacetophenone and *trans*‐4‐phenyl‐3‐buten‐2‐one) were transformed to their (*R*)‐configured alcohols with high conversions, proving the applicability of the system. In another publication, the authors compared different immobilization strategies and observed high space time yields (STYs) of > 450 g·L^−1^·h^−1^ and a continuous production for more than six days [103]. Additionally, the scalability of the hydrogel microreactors for semipreparative applications were recently demonstrated by ‘numbering up’ (up to six modules, continuous flow running for more than eight days) [104].

Bitterwolf et al. also followed the all‐enzyme hydrogel approach based on the ST/SC system [105]. An imine reductase GF3546 from *Streptomyces sp*. was used for the reduction of 3,4‐dihydroisoquinoline to the corresponding amine in an all‐enzyme hydrogel loaded microreactor (Figure 2(iii)). Excellent conversion rates were maintained for up to 40 hours of continuous operation and a maximum STY of 150 g·L^−1^·day^−1^ at 100 μL·min^–1^ was achieved.

Mittmann et al. expanded the self‐assembling all‐enzyme hydrogel methodology to phenolic acid decarboxylases (PADs) [106].The authors reported a continuous flow synthesis of *para*‐hydroxystyrene from *para*‐coumaric acid for more than 10 hours with conversions ≥98% and STY of 57.7 g·L^−1^·day^−1^ using a homodimeric PAD obtained from *Enterobacter* sp. (Figure 2(iv)). For the fabrication of the hydrogels, SC‐ and ST‐tagged PAD variants were created. In addition, the rheological behavior of the hydrogel materials was altered by the modulation of the degree of crosslinking.

Menegatti et al. immobilized whole, permeabilized *Saccharomyces cerevisiae* cells in hydrogels and used it as a hydrogel film in a continuous flow reactor [107]. The cellular fumarase catalyzed the conversion of fumaric acid into l‐malic acid (Figure 2(v)). For enzyme immobilization, the authors investigated hydrogels composed of monomers sodium alginate and PVA as well as calcium chloride and boric or phenylboronic acid as crosslinking agents. Up to 72% of retained fumarase activity was found. A two‐plate microreactor was also constructed resulting in a STY of 2.86 g·L^−1^·h^−1^ with no activity loss during seven days of continuous operation.

Maier et al. reported on the production of flow‐reactor cartridges by 3D printing with thermostable enzymes [108]. The authors describe the use of thermostable enzymes in an agarose hydrogel matrix for the direct ink writing process, in a standard, syringe‐based extrusion printer. An esterase (EstII) or an ADH, from thermophilic organism *Alicyclobacillus acidocaldarius*, were dissolved in liquid agarose, used for the 3D printing of different scaffolds in diverse shapes and sizes. The activity remained constant for the EstII enzyme for 240 min in the printed, hardened agarose samples. Additionally, the authors printed a thermostabilized mutant of a ketoisovalerate decarboxylase (KIVD_mutant) from mesophilic organism *Lactococcus lactis* in agarose. Printed ADH discs and KIVD_mutant discs were combined in a flow setup and an enzymatic cascade was performed in continuous flow to produce isobutanol from ketoisovalerate (Figure 2(vi)).

Yoon et al. co‐immobilized an ene‐reductase from *Thermus scotoductus* SA‐01 (*Ts*OYE) and rose bengal (RB, a light‐harvesting dye) together in alginate hydrogel capsules [109]. With these hydrogel beads, the authors performed a cofactor‐free asymmetric reduction of 2‐methylcyclohexenone to enantiopure (*R*)‐2‐methylcyclohexanone with high enantioselectivities (> 99%) and a maximal conversion of 70.4% (Figure 2(vii)). The authors also found an increase of the robustness of the enzyme with respect to heat (up to 60°C), and aqueous solutions (50% *v*/*v*) of chemical denaturants like dimethylformamide, dimethyl sulfoxide, ethanol, or 1‐propanol.

Schmieg et al. studied the immobilization of β‐galactosidase from *Aspergillus oryzae* (β‐Gal) in PEGDA hydrogel materials [110]. The concentration of enzyme in the hydrogels was 2.5% *w*/*w* and a pneumatic extrusion‐based 3D printer was used to print defined 3D lattice structures. UV‐light was used to harden the hydrogels with the help of the initiator 2‐hydroxy‐4′‐(2‐hydroxyethoxy)‐2‐methylpropiophenone. A standard β‐galactosidase activity assay was performed (*ortho*‐nitrophenyl‐β‐d‐galactopyranoside to galactose and *ortho*‐nitrophenol, see also Figure 2(viii), a) and only minor intrinsic activity loss was observed when the enzyme was incubated in the hydrogel components. However, only 7–10% of entrapped enzyme activity was found when compared to the free enzyme. Mass transfer limitations were calculated to an observable Thiele modulus (*ø*) of more than 20 with an effective diffusivity within the hydrogel of about 3·10^–12^ m^2^·s^–1^. In another study, the concept was broadened to the enzymes benzoylformate decarboxylase from *Pseudomonas putida* (BFD) and *Lb*ADH [111]. Again, the enzymes were immobilized in PEGDA hydrogel materials and 3D‐printed as hydrogel lattices. The lattices were fitted into 3D‐printed reactor housings and operated at constant flow. A continuous product formation could be observed over a period of 72 h for all four enzymatic systems (Figure 2(viii), a–c). Once again, the authors observed mass transport limitations, the effectiveness factor calculated was about 6–9% for those with the greatest reaction rates and up to 14% for the smallest reacting rates.

Simon et al. entrapped five different enzymes in a hydrogel matrix as hydrogel/enzyme dots composed of PEGDA, 2‐(dimethylamino)ethyl methacrylate, and HEMA [112]. Two separate tri‐enzymatic cascade reactions were carried out in continuous flow. For the first cascade, β‐galactosidase from *Aspergillus oryzae* (β‐Gal), glucose oxidase from *Aspergillus niger* (GOx) and HRP were used whereas for the second cascade, phospholipase D from *Streptomyces chromofuscus* (PLD), choline oxidase from *Alcaligenes* sp. (ChOx), and HRP were used. Each cascade produces 2,2′‐azino‐bis(3‐ethylbenzothiazoline‐6‐sulfonic acid) (ABTS, Figure 2(ix)). The 350 μm diameter enzyme hydrogel dots were covalently bonded to planar glass PDMS by UV‐initiated in situ polymerization. A constant reaction rate for a period of at least 15 h of usage were observed. In another study, the authors developed a new technique for the simultaneous photostructuring of PEGDA‐based hydrogels in the μm scale. PDMS‐on‐glass microfluidic devices were yielded with separated reaction chambers [113]. The tri‐enzymatic cascade in Figure 2(ix) (left) was carried out as a proof‐of‐concept.

With respect to hydrogel/enzyme dots, the work of Thiele and co‐workers should also briefly be highlighted in this review. The authors use synthetic and natural monomers and modified hyaluronic acid microgels for enzymatic synthesis [114, 115].

Junshi Moriyama et al. entrapped bovine carbonic anhydrase (BCA) into calcium alginate hydrogel beads with the help of liposomes (BCALs) [116]. Using the BCALs, 98.7 ± 0.2% of the liposome‐enzyme‐adduct was entrapped compared to 27.2 ± 4.1% when the free BCA was used. The authors attribute the significantly lower entrapment efficiency to a passage of free BCA through the alginate matrix. Furthermore, the BCAL‐beads were placed in a column for continuous flow hydrolysis of 1.0 mmol·L^–1^ *para*‐nitrophenyl acetate for 1 h.

Grollmisch et al. immobilized liquid lipase from *Candida antarctica* (CalB) in pILs through encapsulation [69]. To be more specific, the authors used VEtImBr and N,N’‐methylenebisacrylamide (BisA), ammonium peroxydisulfate and N,N,N’,N’‐Tetramethylethylendiamin (TEMED) (Figure 2(x), left) for the synthesis of the hydrogel. The kinetic resolution of *rac*‐1‐phenylethanol with vinyl acetate was studied (Figure 2(x), right) and nonpolar solvents, including *n*‐heptane and *n*‐dodecane were used as reaction media. Nearly full conversion and high catalytic activities were achieved, and the encapsulated lipase was easily recovered from the reaction mixture and reused for ten cycles.

To generalize the applications of immobilized hydrogel‐based enzymes, it can be noted that recently the focus has been on the immobilization strategy and application in specific chemical reactions as a catalyst either as a free bulky particle or as a reactor (or parts thereof). The easy upscaling procedure by numbering up the all‐enzyme hydrogel microreactors and the high space time yields being achieved are promising for future developments in this field. Furthermore, we reviewed the easy immobilization of whole permeabilized cells in hydrogels for the use in continuous flow reactors. Eventually, the use of hydrogels enables the creation of new enzyme‐containing microreactors and/or random column packing materials by 3D printing improving the handling of enzymes, their reusability, and robustness. Nevertheless, mass transport limitations may reduce the overall enzyme's efficiency and must be considered when immobilizing enzymes in hydrogels.

## 3 CONCLUSIONS AND FUTURE PERSPECTIVES

Within this review, our main aim was to cover recent developments in design and development of hydrogel‐based immobilized enzymes and their applications for chemical synthesis. The applications highlighted included both batch and continuous operations. We outlined the drivers behind the rise of this key emerging field: (i) Sustainable and biodegradable materials – considering natural building blocks, (ii) the biocompatibility of many hydrogel materials, (iii) their inherent high water absorption capacity, and (iv) the precision in the control of the production of the hydrogel materials especially thanks to 3D printing technology. Although a few exceptions might exist, mass transport limitations remain challenging and detailed systematic analyses are still missing in the field to a major extent.

We think that future research will most likely concentrate on further developments of novel hydrogel materials for enzyme immobilization and their biocatalytic applications. Particularly, we expect a strong increase in the field of 3D‐printed enzymes being immobilized in hydrogel materials. This might also boost the research field in flow biocatalysis [46], for single‐step or multi‐step catalytic systems. More studies are expected for responsive and self‐healing gels, for their use in enzymatic synthesis [117, 118, 119], while enzymatic reactions can be easily monitored and controlled with the changes/responses detected. In this context, the 4D concept has to be here highlighted which means that the 3D‐printed enzyme hydrogels can show different characteristics over time, being the fourth dimension [120]. We see advantages for the future in the ongoing development of new photoinitiator systems. Since many early initiator systems work with short wavelengths, there is a risk that the proteins will be damaged by UV light and that the activity of the enzymes will be impaired. In addition, low penetration and thus lower film thickness is usually achieved. Therefore, photoinitiator systems with improved absorption at longer irradiation wavelengths have been one of the main focuses of 3D photopolymerization technologies [120].

We also predict that researchers will not only use ionic liquids (ILs) and deep eutectic solvents (DESs) as non‐conventional media for biocatalysis [121], but also apply the polymerized forms of those solvents as hydrogels for enzymatic synthesis. Although the first examples of polymerized ILs have been published only recently, to the best of our knowledge, polymerized DESs have not yet been reported in the literature. Additionally, we expect an increase in interdisciplinary applications, like the use of hydrogel films in bioelectrocatalytic NADPH generation [122]. Overall, the enormous potential of hydrogels for enzyme immobilization is there to be explored in the future.

## CONFLICT OF INTEREST

The authors have declared no conflict of interest.

## Data Availability

Data sharing is not applicable to this article as no new data were created or analyzed in this study.
